# Comparison of multifidus degeneration between scoliosis and lumbar disc herniation

**DOI:** 10.1186/s12891-022-05841-5

**Published:** 2022-09-30

**Authors:** Xianzheng Wang, Huanan Liu, Weijian Wang, Yapeng Sun, Fei Zhang, Lei Guo, Jiaqi Li, Wei Zhang

**Affiliations:** Department of Spinal Surgery, The Third Hospital of Hebei Medical University, 050000 Shijiazhuang, China

**Keywords:** Lumbar disc herniation, Scoliosis, Fatty infiltration, Myogenic disease, Rimmed vacuole

## Abstract

**Objective:**

To assess and compare the pathological and radiological outcomes of multifidus degeneration in scoliosis and lumbar disc herniation patients.

**Methods:**

We performed a retrospective review on 24 patients with scoliosis and 26 patients with lumbar disc herniation (LDH) in the Third Hospital of Hebei Medical University from January 2017 to March2021. The patients were divided into scoliosis group and LDH group according to the treatment. The MRI fatty infiltration rate (FIR) of multifidus and strength of back muscle were calculated to evaluate muscle condition. Multifidus biopsy samples were obtained during surgery in the affected side at L4 or L5 segment in LDH group and on the concavity side of apical vertebrae in scoliosis group. The biopsy fatty infiltration degree (FID) and FIR in two groups, the FIR of affected and unaffected side in LDH group, and the FIR of concavity and convexity side in scoliosis group were compared. The correlation between concavity-convexity FIR difference and cobb angle in scoliosis group, back muscle strength and FIR in LDH group, FID and FIR in both groups was calculated respectively.

**Results:**

The FIR was higher in scoliosis group than in LDH group, higher in concavity side than convexity side in scoliosis group (both *P* < 0.05). The FID was higher in scoliosis group than in LDH group (*P* < 0.05). No significant difference was found between affected and unaffected side in LDH group (*P* > 0.05). There was a positive correlation between concavity-convexity FIR difference and cobb angle, FIR and FID (both *P* < 0.01). There was a negative correlation between back muscle strength and FIR (*P* < 0.01). The biopsy staining results showed that both two groups were found the existence of rimmed vacuoles, nuclear aggregation, and abnormal enzyme activity, indicating that the scoliosis and LDH may be associated with myogenic diseases.

**Conclusion:**

The scoliosis patients showed more serious fatty infiltration than LDH patients and rare pathological findings were found in both diseases.

## Introduction

Scoliosis and lumbar disc herniation (LDH) are common spinal degenerative diseases. In clinical work, severe scoliosis or LDH often requires surgical treatment to correct the spinal curvature or remove the diseased intervertebral disc. The main hypothesis of scoliosis includes asymmetric bone growth dysregulation, genetic disease, genetic factors and connective tissue anomalies [1, 2]. According to previous studies, lumbar disc degeneration is usually accompanied by high fat infiltration of paravertebral muscles and muscle disease can also lead to scoliosis [3, 4]. In general, the etiology of scoliosis and LDH is still controversial.

The muscular atrophy and hypertrophy [5], mitochondrial abnormality [6], fatty involution, and presence of hyaline fibers were found in the paravertebral muscle of the scoliosis [7]. Hsu’s study showed that type 2 A fibers predominated over type 2B in scoliosis patients, which is contrary to the normal paravertebral muscle [8]. Increase of lipid droplets and glycogen particles, dilated sarcoplasmic reticulum [9], and decreased oxidative enzymatic activity [10] indicate that scoliosis is probably associated with muscle lesions. In addition, similar manifestations were also observed in the paravertebral muscles of patients with lumbar disc herniation [11, 12].

Scoliosis patients are often accompanied by paravertebral muscle fat infiltration and muscle strength decline. We are concerned that patients with fat infiltration may not be able to maintain spinal curvature after surgery due to paravertebral muscle weakness. The weakness of muscle strength may accelerate the degeneration of the spine, which may induce the occurrence of LDH. Multifidus is the main source of lumbar stability [13]. Muscle lesion is one of the hypotheses of scoliosis, which is also a risk factor of LDH. Therefore, we compared the paravertebral muscle of the two diseases, trying to find out the consistency and difference and whether early muscle treatment can avoid the further development of spinal degeneration, such as gene sequencing and early diagnosis of rare muscle diseases.

## Materials and methods

All procedures performed in studies involving human patients were in accordance with the 1964 Helsinki declaration. The latter waived written informed consent because of the retrospective nature of our study.

### Patient population

**Fig. 1:**
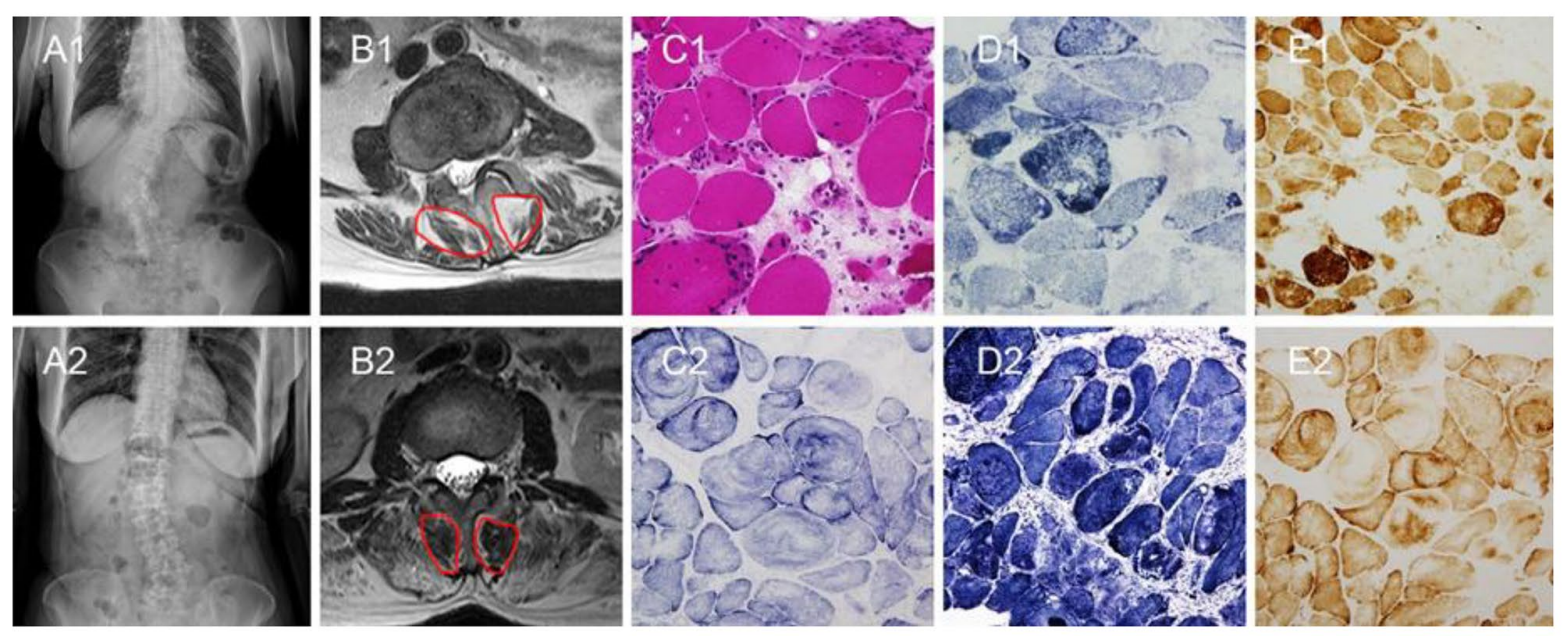
Two cases of scoliosis. Patient 1: A1-E1. Patient 2: A2-E2. (**A1**) The X-ray radiograph shows the degree of scoliosis. (**B1**) The T2-weighted axial MRI at L2 shows the multifidus morphology. (**C1**) The hematoxylin-eosin staining shows the rimmed vacuole. (**D1**) The NADH dehydrogenase staining shows the NADH dehydrogenase activity decreased. (**E1**) The Cytochrome C oxidase enzymes staining shows the Cytochrome C oxidase enzymes activity decreased. (**A2**) The X-ray radiograph shows the degree of scoliosis. (**B2**) The T2-weighted axial MRI at L1 shows the multifidus morphology. (**C2**) The Succinate dehydrogenase staining shows severe activity disorder of succinate dehydrogenase. (**D2**) The NADH dehydrogenase staining shows severe activity disorder of NADH dehydrogenase. (**E2**) The Cytochrome C oxidase enzymes staining shows severe activity disorder of Cytochrome C oxidase enzymes

**Fig. 2:**
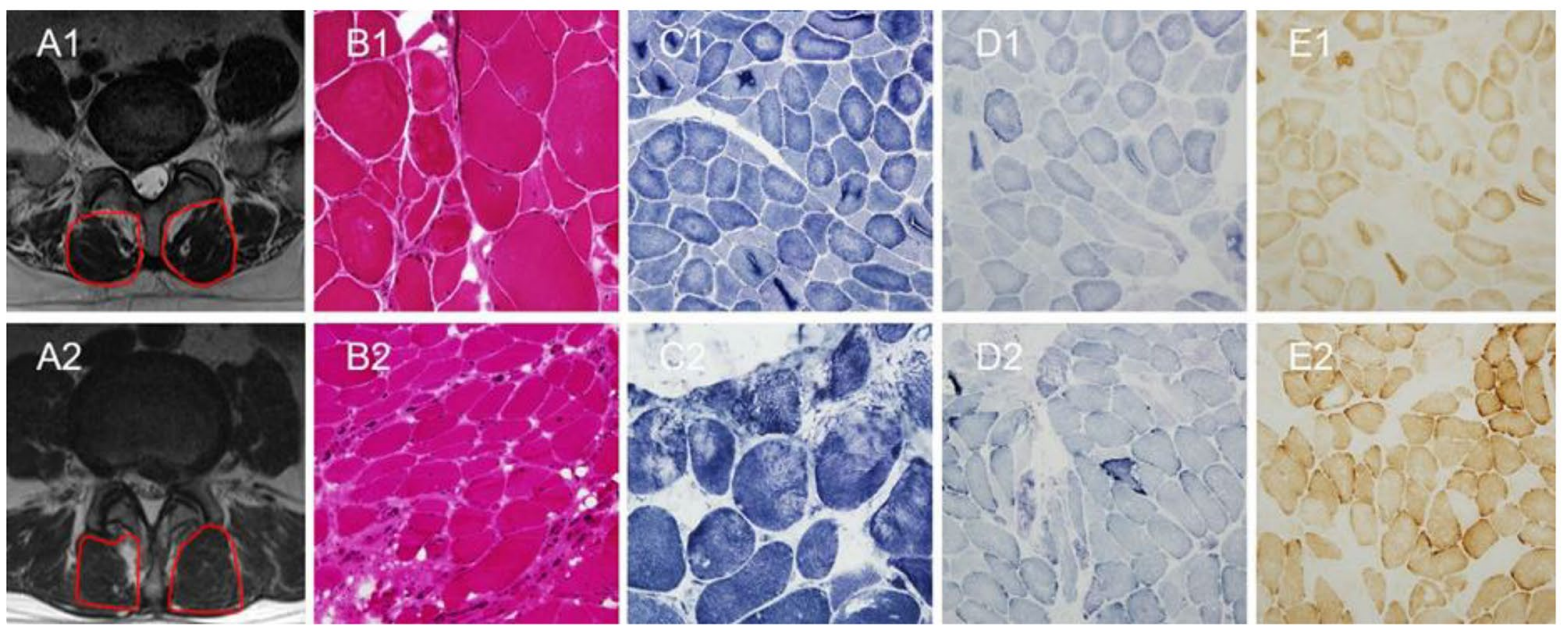
Two cases of lumbar disc herniation. Patient 1: A1-E1. Patient 2: A2-E2. (**A1**) The T2-weighted axial at L5 MRI shows the multifidus morphology. (**B1**) The hematoxylin-eosin staining shows the rimmed vacuole. (**C1**) The NADH dehydrogenase staining shows the NADH dehydrogenase activity decreased and rimmed vacuole. (**D1**) The Succinate dehydrogenase staining shows shows the Succinate dehydrogenase staining activity decreased. (**E1**) The Cytochrome C oxidase enzymes staining shows the Cytochrome C oxidase enzymes activity decreased. (**A2**) The T2-weighted axial MRI at L4 shows the multifidus morphology. (**B2**) The hematoxylin-eosin staining shows severe fatty infiltration. (**C2**, **D2**, **E2**) The hematoxylin-eosin staining, NADH dehydrogenase staining, Cytochrome C oxidase enzymes staining shows myofibrillar network disorder

24 patients with scoliosis and 26 patients with lumbar disc herniation (LDH) in the Third Hospital of Hebei Medical University from January 2017 to March 2021 were included (Figs. 1 and 2). All patients were treated by the same surgeon.

Power analysis was based on data of previous studies on the infiltration rate of LDH and scoliosis [14, 15] and showed that at least 23 patients were needed in each group to reach a high statistical power (> 0.8). After applying inclusion and exclusion criteria we enrolled 50 patients in our analysis.

Inclusion criteria for this study included (1) patients diagnosed with LDH or thoracolumbar scoliosis with ineffective conservative treatment for more than 3 months; (2) responsible segments were confirmed by CT and MRI; (3) Patients with pathological biopsy during operation. Exclusion criteria included (1) fracture, tumor, tuberculosis, spinal cord injury, (2) severe lung, heart disease or other surgical contraindications, (3) spinal operation history.

### Radiographic and strength analysis

All patients underwent MRI and X-ray examinations before surgery. The MRI images were obtained in the same 1.5T device (Avanto, Siemens, Germany) by a fast spin echo pulse sequence and the display field of view was 350 × 350 mm. Slice thickness was 4 mm. The MRI radiologists were blind to this study.

### Subcutaneous fat tissue thickness (SFTT, L1-L2)

The subcutaneous fat tissue thickness (SFTT, L1-L2) was measured as the vertical distance from the tip of spinous process to the skin on axial T2-weighted lumbar spine MRI. Compared with BMI, SFTT has a better correlation with the overall health status of patients. In addition, SFTT at upper lumbar levels (specifically L1-L2) was more valuable in predicting lumbar degeneration [16].

### Fatty infiltration rate (FIR)

The fatty area (FA) and the cross section area (CSA) of multifidus in axial MRI were analyzed with Image-pro Plus software (version 6.0, Media Cybernetics, Inc., USA). The region of interest was drawn by outlined the muscle fascia. The FIR was calculated as follows: FA/CSA. The FIR of affected/unaffected side in LDH group and convex/concave side in scoliosis group were calculated respectively.

### Cobb angle

The Cobb angle was calculated as follows: choose the most tilted vertebrae above and below the apex of the curve and the angle between intersecting lines drawn perpendicular to the top of the top vertebrae and the bottom of the bottom vertebrae is the Cobb angle.

### Lumbar dorsal strength

The method for measuring the strength of the lumbodorsal muscles was as follows. The patient was in the prone position, with the lower limbs straight and the arms on both sides of the trunk, and the abdomen was padded with a pillow to lift the chest as much as possible and maintain the maximum flexion of the cervical spine, so as to reduce the lumbar lordosis angle and the load on the lumbar spine. The length of time the patient held the posture was recorded.

## Histological analysis

### Multifidus biopsy

Muscle biopsy specimens (0.5 cm diameter, 1.0 cm length) were collected from the multifidus in the affected side at L4 or L5 segment in LDH group and on the convex side of apical vertebrae in scoliosis group during surgery. The muscle biopsy specimens were frozen in isopentane cooled in liquid nitrogen, and 7 μm cryostat sections were made using an EM UC ultrathin section machine (Leica Microsystems, Mannheim, Germany), and were used for the subsequent histochemistry and immunofluorescence staining experiments. Morphological analysis of the muscle was performed using the following routine histological stains: Hematoxylin and eosin, modified Gomori trichrome, NADH‑tetrazoliumreductase, succinate dehydrogenase, adenosine monophosphate deaminase and cytochrome c oxidase to assess enzymatic activity, adenosine triphosphatase at pH 9.8, 4.3 and 4.6 to assess muscle fiber distribution, acid phosphatase, and oil red O staining to assess fatty deposition (all Shanghai Bioleaf Biotech Co., Ltd., Shanghai, China). After staining the muscle specimens were visualized, and the images were captured, using a BX51 confocal scanning laser microscope (Olympus Corporation, Tokyo, Japan [17].

### Fatty infiltration degree (FID)

Analysis was made by two experienced pathologists, blind to the MRI results and this study. We chose FID as one of the evaluation indicators of fat infiltration and set the grade [15]. The FID was evaluated as follows: 0 is defined as “none, normal muscle”, 1 as “scarce fatty infiltration, with up to 25% of the muscle fibers involved”, 2 as “mild changes, with light infiltration, with 26–50% of the muscle fibers showing fatty infiltration”, 3 as “moderate alterations, with 51–75% of tissue infiltrated with fat”, and 4 as “severe changes, with more than 76% of infiltration”. The discordance in the analysis was solved in a consensus conference.

### Statistical methods

The Student’s t test, Wilcoxon nonparametric test, Paired t test, Pearson Chi Square and Fisher’s exact test were used when appropriate to test the significance of the differences within and between the groups. The Pearson correlation coefficient, Spearman’s rank correlation coefficient were used to test the correlation between the two factors. We analyzed the date with the SPSS (SPSS Statistics 25.0, IBM Inc., Chicago, IL, USA). Statistical significance was set at P < 0.05. All the results are given as mean values ± standard deviation.

## Results

### Baseline and clinical outcomes

The baseline characteristics of the patient population were summarized in Table 1. A total of 44 patients (20 men) were involved for analysis, in which 24 scoliosis patients and 26 LDH patients were included. The average age at baseline was 52 (scoliosis group) and 51(LDH group). There were no significant differences in sex, age and body mass index (*P* > 0.05).

**Table 1:** Patient demographic and radiographic data

Variable	Scoliosis	LDH	***P***-value
	(n = 24)	(n = 26)	
Age, years	52.27 ± 3.96	51.54 ± 3.06	0.465^†^
Sex (male, female)	9, 15	11, 15	0.779^§^
Body mass index (kg/m^2^)	21.01 ± 0.62	21.34 ± 0.95	0.120^‡^
SFTT (mm)	13.43 ± 7.04	15.42 ± 7.14	0.329^†^
Total Fatty area (pixels)	1808.47 ± 96.17	1497.39 ± 101.14	< 0.001^†^
Total CSA (pixels)	7995.05 ± 229.11	9976.49 ± 235.97	< 0.001^‡^
Total FIR (%)	22.67 ± 1.37	15.00 ± 1.09	< 0.001^‡^

Data were presented as mean ± SD or as shown

LDH, lumbar disc herniation; CSA, cross-sectional area; FA, fatty area; FIR, fatty infiltration rate; SFTT, subcutaneous fat tissue thickness

^†^, Student t test; ^‡^, Wilcoxon nonparametric test; ^§^, Pearson Chi-square test

### Radiographic outcomes

The radiographic outcomes of the patients are shown in Table 2. The FIR was higher in scoliosis group than in LDH group, higher in concavity side than convexity side in scoliosis group (both *P* < 0.05). No significant difference was found between affected and unaffected side in LDH group (*P* > 0.05). There was a positive correlation between concavity-convexity FIR difference and cobb angle, FIR and FID (both *P* < 0.01). There was a negative correlation between back muscle strength and FIR (*P* < 0.01).

**Table 2:** Radiographic evaluation of fatty infiltration

	Scoliosis			LDH		
Scores	Concavity	Convexity	*P*-value	Affected side	Unaffected side	*P*-value
Fatty area (pixels)	1006.84 ± 93.38	807.11 ± 42.51	< 0.001^¶^	714.52 ± 106.46	703.31 ± 116.97	0.683^¶^
CSA (pixels)	4008.72 ± 210.25	4034.11 ± 214.83	0.109^¶^	4996.10 ± 178.93	5015.88 ± 191.29	0.158^¶^
FIR (%)	25.07 ± 1.11	20.01 ± 0.41	< 0.001^¶^	14.25 ± 1.63	14.02 ± 2.24	0.668^¶^

Data were presented as mean ± SD.

LDH, lumbar disc herniation; CSA, cross-sectional area; FA, fatty area; FIR, fatty infiltration rate;

^¶^, Paired t test

### Histological outcomes

The histological outcomes of the patients are shown in Tables 3 and 4. The FID was higher in scoliosis group than in LDH group (*P* < 0.05). The biopsy staining results showed that both two groups were found the existence of rimmed vacuoles and abnormal enzyme activity, indicating that the scoliosis and LDH may be associated with myogenic diseases, though no significant difference were found between the two groups.

**Table 3:** Histological evaluation of fatty infiltration

	Scoliosis	LDH
Scores	(n = 24)	(n = 26)
None (0)	0	0
Scarce (1)	5	8
Mild (2)	7	11
Moderate (3)	5	6
Severe (4)	7	1
*P*-value	< 0.001^‡^	

LDH, lumbar disc herniation

^‡^, Wilcoxon nonparametric test

**Table 4:** Pathological features

Variable (n, %)	Scoliosis	LDH	***P***-value
	(n = 24)	(n = 26)	
Rimmed vacuoles	5 (20.8%)	7 (26.9%)	0.614^§^
Type I fiber predominance	0	2 (7.7%)	0.491^$^
Nuclear aggregation	2 (8.3%)	0	0.469^$^
Abnormal enzyme activity			
Adenosine monophosphate	0	1 (3.8%)	1^$^
Adenosine triphosphate cyclase	2 (8.3%)	0	0.225^$^
Acid phosphatase	0	1 (3.8%)	1^$^
NADH dehydrogenase	1 (4.2%)	2 (7.7%)	1^$^
Cytochrome C oxidase staining enzymes	1 (4.2%)	1 (3.8%)	1^$^
Succinate dehydrogenase	0	1 (3.8%)	1^$^

Data were presented as number (percentage)

^§^, Pearson Chi-square test; ^$^, Fisher’s exact test

### Correlation analysis

**Fig. 3:**
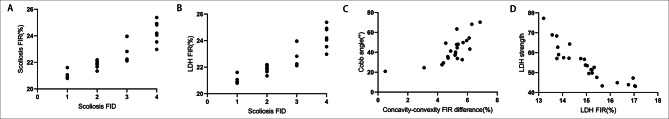
(**A**) Correlation between the FIR and FID in the scoliosis group. (**B**) Correlation between the FIR and FID in the LDH group. (**C**) Correlation between the cobb angle and concavity-convexity FIR difference in the scoliosis group. (**D**) Correlation between the lumbar dorsal muscle strength and FIR in the LDH group. FIR, fatty infiltration rate; FID, fatty infiltration degree; LDH, lumbar disc herniation

The correlation analysis outcomes of the patients are shown in Table 5; Fig. 3. Cobb angle showed positive correlation (Pearson’s r = 0.929, *p* < 0.01) with the concavity-convexity FIR difference. LDH strength showed positive correlation (Pearson’s r = -0.887, *p* < 0.01) with the LDH FIR. There was a significant correlation between scoliosis FIR and scoliosis FID (Spearman’s rank correlation coefficient 0.929, *p* < 0.01). There was a significant correlation between LDH FIR and LDH FID (Spearman’s rank correlation coefficient 0.915, *p* < 0.01).

**Table 5:** The correlation analysis.

Group		***r***	***P***-value
Scoliosis FIR	22.67 ± 1.37%	*r*_*s*_	
Scoliosis FID	2.58 ± 1.13	0.929	< 0.01
LDH FIR	15.00 ± 1.09%	*r*_*s*_	
LDH FID	2.00 ± 0.84	0.915	< 0.01
Cobb angle	43.09 ± 12.85	*r*	
Concavity-convexity FIR difference	5.05 ± 1.24%	0.721	< 0.01
LDH strength	54.45 ± 8.70	*r*	
LDH FIR	15.00 ± 1.09%	-0.887	< 0.01

Data were presented as mean ± SD.

*r*_*s*_: Spearman’s rank correlation coefficient; *r*: Pearson correlation coefficient.

## Discussion

### Rimmed vacuoles and chronic myopathy changes

In 1973, Dubowitz and Brooke first named “rimmed vacuoles” as the pathological feature observed in muscular dystrophy patient, which refers to the pathological phenomenon that small vacuoles or cracks in the cytoplasm of muscle fibers observed through the modified Gomori trichrome staining, with red stained particles in the inner edge. Generally speaking, rimmed vacuoles are usually found in myogenic diseases, such as distal myopathy, sporadic inclusion body myositis and myofibrillar myopathy, which are closely related to congenital gene mutation [18–21]. In this study, we found that a high proportion of rimmed vacuoles appeared in LDH and scoliosis patients and no significant difference was found between the two groups. According to the past literature, the rimmed vacuoles were mostly associated with congenital myogenic diseases, but there were no reports on the high proportion of rimmed vacuoles in LDH and degenerative scoliosis. The rimmed vacuoles are composed of autophagic vacuoles and myeloid bodies [22], indicating that autophagy occurs in muscle fibers, which is the main cellular pathway for degradation of expired proteins and organelles in eukaryotic cells and the formation of autophagic vacuoles in myopathy is considered to be a secondary reaction due to abnormal lysosomal function [23, 24]. There are several conjectures about the formation of rimmed vacuoles, such as oxidative stress response, excess of substrate in normal lysosomes and secondary response to endoplasmic reticulum stress [25]. We speculate that the rimmed vacuoles may be related to myogenic diseases without obvious symptoms or aseptic inflammation leads to oxidative stress in muscles, which leads to the appearance of rimmed vacuoles. The relationship between rimmed vacuoles, LDH and degenerative scoliosis can be further explored, so as to find an early treatment of myogenic changes or oxidative stress which can avoid subsequent degeneration of spinal bone structure and intervertebral disc.

In addition, we also observed the occurrence of type I fiber predominance, nuclear aggregation and abnormal enzyme activity in patients, which suggested that there were chronic myogenic diseases in the patients at the same time. Succinate dehydrogenase staining is the most sensitive method to reflect the abnormal accumulation of mitochondria in muscle cells and vascular wall. There was a positive correlation between succinate dehydrogenase activity and muscle oxygen delivery ability, oxygen transport from interstitial space to the core of the muscle fibers, and oxidative capacity [26]. Cytochrome c oxidase is the terminal complex of 13 subunits of mitochondrial electron transport chain, the staining of which is an important method to check the abnormality of mitochondrial electron transport chain, reflect whether the metabolism in muscle cells is normal, and can distinguish the types of muscle fibers at the same time [27]. NADH is a coenzyme that provides redox ability for mitochondria to produce ATP, and NADH staining can clearly observe the distribution of type I and type II muscle fibers and judge the pathological phenomena such as muscle cell metabolism and myofibril arrangement [28]. In our experiment, there was no significant difference in biopsy enzyme activity between the two groups. However, the results showed different degrees of enzyme activity disorder and pathological phenomena, suggesting that the chronic myopathy may exist in patient.

### Biopsy and MRI in the diagnose in the fatty infiltration

Multifidus can prevent vertebral rotation dislocation, maintain lumbar lordosis, increase lumbar segmental tension, and reduce the movement between lumbar segments [29]. However, fat infiltration of multifidus muscle is common in spinal degenerative diseases [30, 31]. Usually, the fat infiltration rate of multifidus is measured by MRI, but pathological biopsy of multifidus is more intuitive and accurate in the observation. It is very important to detect the degree of fat infiltration of multifidus. The targeted strength training and daily life behavior correction of patients can be carried out according to the test results, which is of great significance to enhance the strength of multifidus muscle and delay spinal degeneration. As a common non-invasive examination, the accuracy of MRI in judging fat infiltration is of great significance to clinical work. In this study, the results of biopsy and MRI were compared and the results are consistent, insistent with the Wajchenberg’s study [32], which proved that MRI is accurate in the diagnosis of fat infiltration. To make the influence of human factors on the calculation results further excluded, Li developed an measurement system for automatic segmentation of multifidus and erector spinae in MRI images based on deep neural network [33] and Bahar used custom written Matlab software and two-term Gaussian model to calculated fat signal fraction [34], which furtherly avoid human factors on the calculation of fat infiltration rate.

### Fat infiltration and lumbar dorsal mulscle strength

Studies have shown that paravertebral muscle atrophy, fat content increased, paraspinal muscle weakness may lead to decreased spinal stability, which is closely related to a variety of lumbar diseases [35, 36]. The muscle strength furtherly decreased after operation due to muscle damage during operation and use of internal fixation instruments [37]. The integrity and vitality of paravertebral muscles play an important role in maintaining the quality of adjacent vertebrae. After bilateral erector spinae muscles were removed, the local vertebral bone mass decreased significantly, suggesting that paravertebral muscle injury may cause vertebral osteoporosis [38]. In patients with osteoporosis, pedicle screw loosening is one of the main complications, and the severe cases may lead to failure of operation [39]. Therefore, preoperative observation of the degree of paravertebral muscle fat infiltration is of great significance for the prediction of postoperative recovery. Preoperative cross sectional area of multifidus is a reliable predictor of postoperative clinical outcomes. Muscle atrophy is associated with poor prognosis [40]. In this study, there was a negative correlation between fat infiltration and strength. For patients with symptomatic lumbar disease, it may be difficult to measure the strength of paravertebral muscles reliably, for the back pain and discomfort may influnce the accuracy of assessment. At the same time, the upper body mass and willpower will also affect the test results. However, the methods for assessing strength had been proved high reliability, reproducibility, and safety [41] and has been widely used in clinical evaluation of low back muscle strength [42].

### Convex and the concave sides

In our study, fat infiltration on the concave surface was more severe. There was a positive correlation between the cobb angle and difference of fatty infiltration rate, which indicated that the higher the cobb angle, the greater the difference between the two sides of the fatty infiltration rate. In scoliosis patients, because of the deformity of spinal curvature, the asymmetry of bilateral paravertebral muscles is common, which also increases with the progress of Cobb angle [43]. Paravertebral muscle imbalance is one of the most important factors in the occurrence and development of scoliosis [44] and muscle atrophy is associated with increased fat infiltration [45]. In Khosla’s study, significant morphological changes were found at the juncture of tendon sheath and discontinuous structural defects appeared on the sarcolemma of muscle fibers, which is associated with increased fatty infiltration [46]. Stetkarova et al. showed that the proportion of type I fiber increased on the convexity side and decreased on the concave side [47]. Our results are insistent with the previous studies, which supports the theory of muscle imbalance to a certain extent [48].

### Fatty infiltration in LDH and scoliosis

Intramuscular fat are associated with disuse, sex steroid deficiency, glucocorticoid treatment, and altered leptin signaling [49]. Furthermore, the accumulation of iadipocyte and ntramuscular lipid in skeletal muscle are linked to the loss of muscle strength and increased mortality in the elderly. In this study, the degree of fat infiltration in scoliosis group was significantly higher than that in LDH group. LDH can be caused by denervation and disuse caused by pain, and the abnormal structure of scoliosis is an important cause of paraspinal muscle changes. Parkkola et al. [50]found that paravertebral muscle atrophy was obvious when cross section area was not reduced, and the atrophic muscle was replaced by fat and fibrous tissue. Kjaer et al. found that when unilateral lumbar nerve root was compressed and injured, bilateral multifidus muscle had increased fat infiltration [51], which was explained by Hodges that the mechanism of dorsal branch reflex inhibition was stopped [52]. The mechanism of fatty infiltration includes: (1) accumulation of intramyocellular lipid, which was associated with insulin insensitivity, inflammation, and functional deficits [53]; (2) accumulation of adipocyte [54]. Fibro/adipogenic progenitor, a kind of precursor cells, can differentiate into adipocytes or myofibroblasts in chronic injury environment, which explain that fibrosis is a concomitant phenomenon in the process of muscle regeneration [55].

### Unresolved issues

We only found the presence of rimmed vacuoles, but we did not find the reason for rimmed vacuoles appearing in LDH and scoliosis patients, which can be further studied by immunohistochemistry, gene sequencing and family map analysis.

## Limitations

First, the T2 MRI cannot effectively identify the muscle inflammation and fatty infiltration, which had an impact on our experimental results. The fluid attenuated inversion recovery sequence can be used to identify inflammation in further study. Second, the strength assessment method we used is not of high accuracy. Though the strength assessed by the lumbar extension machine may have higher accuracy than the method we used, the high price, complex operation make it mostly used for sports research and training at present.

## Conclusion

The scoliosis patients showed more serious fatty infiltration than LDH patients and rare pathological findings were found in both diseases.

## Data Availability

The datasets used or analyzed during the current study are available from the corresponding author on reasonable request.

## Code Availability

The custom-written software used in this paper is available via reasonable request from the corresponding author.

## References

[1] de Sèze M, Cugy E (2012). Pathogenesis of idiopathic scoliosis: a review. Annals of physical and rehabilitation medicine.

[2] Yaman O, Dalbayrak S (2014). Idiopathic scoliosis. Turkish Neurosurg.

[3] Vialle R, Thévenin-Lemoine C, Mary P (2013). Neuromuscular scoliosis. Orthop Traumatol Surg research: OTSR.

[4] Teichtahl AJ, Urquhart DM, Wang Y, Wluka AE, O’Sullivan R, Jones G, Cicuttini FM (2016). Lumbar disc degeneration is associated with modic change and high paraspinal fat content - a 3.0T magnetic resonance imaging study. BMC Musculoskelet Disord.

[5] Kim H, Lee CK, Yeom JS, Lee JH, Cho JH, Shin SI, Lee HJ, Chang BS (2013). Asymmetry of the cross-sectional area of paravertebral and psoas muscle in patients with degenerative scoliosis. Eur spine journal: official publication Eur Spine Soc Eur Spinal Deformity Soc Eur Sect Cerv Spine Res Soc.

[6] Li Z, Shen J, Liang J (2015). Scoliosis in mitochondrial myopathy: case report and review of the literature. Medicine.

[7] Wajchenberg M, Martins DE, Luciano Rde P, Puertas EB, Del Curto D, Schmidt B, Oliveira AB, Faloppa F (2015). Histochemical analysis of paraspinal rotator muscles from patients with adolescent idiopathic scoliosis: a cross-sectional study. Medicine.

[8] Hsu JD, Slager UT, Swank SM, Robinson MH (1988). Idiopathic scoliosis: a clinical, morphometric, and histopathological correlation. J Pediatr Orthop.

[9] Low WD, Chew EC, Kung LS, Hsu LC, Leong JC. Ultrastructures of nerve fibers and muscle spindles in adolescent idiopathic scoliosis. Clinical orthopaedics and related research 1983(174):217–221. 6219851

[10] Kumano K (1980). Congenital non-progressive myopathy, associated with scoliosis–clinical, histological, histochemical and electron microscopic studies of seven cases. Nihon Seikeigeka Gakkai zasshi.

[11] Cooley JR, Walker BF, Kjaer EMA, Jensen P, Hebert TS (2018). Relationships between paraspinal muscle morphology and neurocompressive conditions of the lumbar spine: a systematic review with meta-analysis. BMC Musculoskelet Disord.

[12] Delisle MB, Laroche M, Dupont H, Rochaix P, Rumeau JL (1993). Morphological analyses of paraspinal muscles: comparison of progressive lumbar kyphosis (camptocormia) and narrowing of lumbar canal by disc protrusions. Neuromuscul disorders: NMD.

[13] Wilke HJ, Wolf S, Claes LE, Arand M, Wiesend A (1995). Stability increase of the lumbar spine with different muscle groups. A biomechanical in vitro study. Spine.

[14] Elysee JC, Lovecchio F, Lafage R, Ang B, Huang A, Bannwarth M, Kim HJ, Schwab F, Lafage V (2021). The relationship of global sagittal malalignment to fatty infiltration in the aging spine. Eur spine journal: official publication Eur Spine Soc Eur Spinal Deformity Soc Eur Sect Cerv Spine Res Soc.

[15] Shi L, Yan B, Jiao Y, Chen Z, Zheng Y, Lin Y, Cao P (2022). Correlation between the fatty infiltration of paraspinal muscles and disc degeneration and the underlying mechanism. BMC Musculoskelet Disord.

[16] Özcan-Ekşi EE, Kara M, Berikol G, Orhun Ö, Turgut VU, Ekşi M (2022). A new radiological index for the assessment of higher body fat status and lumbar spine degeneration. Skeletal Radiol.

[17] Li N, Zhao Z, Shen H, Bing Q, Guo X, Hu J (2018). MYH7 mutation associated with two phenotypes of myopathy. Neurol sciences: official J Italian Neurol Soc Italian Soc Clin Neurophysiol.

[18] Jongen PJ, Ter Laak HJ, Stadhouders AM (1995). Rimmed basophilic vacuoles and filamentous inclusions in neuromuscular disorders. Neuromuscul disorders: NMD.

[19] Niu Z, Pontifex CS, Berini S, Hamilton LE, Naddaf E, Wieben E, Aleff RA, Martens K, Gruber A, Engel AG (2018). Myopathy With SQSTM1 and TIA1 Variants: Clinical and Pathological Features. Front Neurol.

[20] D’Agostino C, Nogalska A, Cacciottolo M, Engel WK, Askanas V (2011). Abnormalities of NBR1, a novel autophagy-associated protein, in muscle fibers of sporadic inclusion-body myositis. Acta Neuropathol.

[21] Lamont PJ, Udd B, Mastaglia FL, de Visser M, Hedera P, Voit T, Bridges LR, Fabian V, Rozemuller A, Laing NG (2006). Laing early onset distal myopathy: slow myosin defect with variable abnormalities on muscle biopsy. J Neurol Neurosurg Psychiatry.

[22] Berardo A, Lornage X, Johari M, Evangelista T, Cejas C, Barroso F, Dubrovsky A, Bui MT, Brochier G, Saccoliti M (2019). HNRNPDL-related muscular dystrophy: expanding the clinical, morphological and MRI phenotypes. J Neurol.

[23] Ii K, Hizawa K, Nonaka I, Sugita H, Kominami E, Katunuma N (1986). Abnormal increases of lysosomal cysteinine proteinases in rimmed vacuoles in the skeletal muscle. Am J Pathol.

[24] Tsuruta Y, Furuta A, Furuta K, Yamada T, Kira J, Iwaki T (2001). Expression of the lysosome-associated membrane proteins in myopathies with rimmed vacuoles. Acta Neuropathol.

[25] Malicdan MC, Noguchi S, Nishino I (2007). Autophagy in a mouse model of distal myopathy with rimmed vacuoles or hereditary inclusion body myopathy. Autophagy.

[26] Degens H, Veerkamp JH (1994). Changes in oxidative capacity and fatigue resistance in skeletal muscle. Int J Biochem.

[27] Lee I, Hüttemann M, Liu J, Grossman LI, Malek MH (2012). Deletion of heart-type cytochrome c oxidase subunit 7a1 impairs skeletal muscle angiogenesis and oxidative phosphorylation. J Physiol.

[28] White AT, Schenk S (2012). NAD(+)/NADH and skeletal muscle mitochondrial adaptations to exercise. Am J Physiol Endocrinol metabolism.

[29] Danneels LA, Vanderstraeten GG, Cambier DC, Witvrouw EE, De Cuyper HJ (2000). CT imaging of trunk muscles in chronic low back pain patients and healthy control subjects. Eur spine journal: official publication Eur Spine Soc Eur Spinal Deformity Soc Eur Sect Cerv Spine Res Soc.

[30] Liu Y, Liu Y, Hai Y, Li G, Liu T, Wang Y (2020). Lumbar lordosis reduction and disc bulge may correlate with multifidus muscle fatty infiltration in patients with single-segment degenerative lumbar spinal stenosis. Clin Neurol Neurosurg.

[31] Shin MH, Ryu KS (2017). MRI-based determination of convex or concave surgical approach for lateral lumbar interbody fusion in lumbar degenerative scoliosis: a retrospective radiographic comparative analysis. J Neurosurg Sci.

[32] Wajchenberg M, Astur N, Fernandes EA, Paredes-Gamero EJ, Luciano RP, Schmidt B, Oliveira ASB, Martins DE (2019). Assessment of fatty infiltration of the multifidus muscle in patients with adolescent idiopathic scoliosis through evaluation by magnetic resonance imaging compared with histological analysis: a diagnostic accuracy study. J Pediatr Orthop Part B.

[33] Li H, Luo H, Liu Y. Paraspinal Muscle Segmentation Based on Deep Neural Network. Sensors (Basel, Switzerland) 2019, 19(12). 10.3390/s19122650 PMC6630766 31212736

[34] Shahidi B, Parra CL, Berry DB, Hubbard JC, Gombatto S, Zlomislic V, Allen RT, Hughes-Austin J, Garfin S, Ward SR (2017). Contribution of Lumbar Spine Pathology and Age to Paraspinal Muscle Size and Fatty Infiltration. Spine.

[35] Shafaq N, Suzuki A, Matsumura A, Terai H, Toyoda H, Yasuda H, Ibrahim M, Nakamura H (2012). Asymmetric degeneration of paravertebral muscles in patients with degenerative lumbar scoliosis. Spine.

[36] Sun D, Liu P, Cheng J, Ma Z, Liu J, Qin T (2017). Correlation between intervertebral disc degeneration, paraspinal muscle atrophy, and lumbar facet joints degeneration in patients with lumbar disc herniation. BMC Musculoskelet Disord.

[37] Lee CS, Kang KC, Chung SS, Park WH, Shin WJ, Seo YG (2017). How does back muscle strength change after posterior lumbar interbody fusion?. J Neurosurg Spine.

[38] Wang XP, Wang SJ, Yan P, Zhu LL, Li MQ, Bian ZY, Tian JW (2016). [Effect of lumbar dorsal muscle injuries on lumbar vertebral bone quality of rat]. Zhonghua yi xue za zhi.

[39] Weiser L, Huber G, Sellenschloh K, Viezens L, Püschel K, Morlock MM, Lehmann W (2017). Insufficient stability of pedicle screws in osteoporotic vertebrae: biomechanical correlation of bone mineral density and pedicle screw fixation strength. Eur spine journal: official publication Eur Spine Soc Eur Spinal Deformity Soc Eur Sect Cerv Spine Res Soc.

[40] Zotti MGT, Boas FV, Clifton T, Piche M, Yoon WW, Freeman BJC (2017). Does pre-operative magnetic resonance imaging of the lumbar multifidus muscle predict clinical outcomes following lumbar spinal decompression for symptomatic spinal stenosis?. Eur spine journal: official publication Eur Spine Soc Eur Spinal Deformity Soc Eur Sect Cerv Spine Res Soc.

[41] Ito T, Shirado O, Suzuki H, Takahashi M, Kaneda K, Strax TE (1996). Lumbar trunk muscle endurance testing: an inexpensive alternative to a machine for evaluation. Arch Phys Med Rehabil.

[42] Simson KJ, Miller CT, Ford J, Hahne A, Main L, Rantalainen T, Teo WP, Teychenne M, Connell D, Trudel G (2017). Optimising conservative management of chronic low back pain: study protocol for a randomised controlled trial. Trials.

[43] Xie D, Zhang J, Ding W, Yang S, Yang D, Ma L, Zhang J (2019). Abnormal change of paravertebral muscle in adult degenerative scoliosis and its association with bony structural parameters. Eur spine journal: official publication Eur Spine Soc Eur Spinal Deformity Soc Eur Sect Cerv Spine Res Soc.

[44] Modi HN, Suh SW, Yang JH, Hong JY, Venkatesh K, Muzaffar N (2010). Spontaneous regression of curve in immature idiopathic scoliosis - does spinal column play a role to balance? An observation with literature review. J Orthop Surg Res.

[45] Jiang J, Meng Y, Jin X, Zhang C, Zhao J, Wang C, Gao R, Zhou X (2017). Volumetric and Fatty Infiltration Imbalance of Deep Paravertebral Muscles in Adolescent Idiopathic Scoliosis. Med Sci monitor: Int Med J experimental Clin Res.

[46] Khosla S, Tredwell SJ, Day B, Shinn SL, Ovalle WK (1980). An ultrastructural study of multifidus muscle in progressive idiopathic scoliosis. Changes resulting from a sarcolemmal defect at the myotendinous junction. J Neurol Sci.

[47] Stetkarova I, Zamecnik J, Bocek V, Vasko P, Brabec K, Krbec M (2016). Electrophysiological and histological changes of paraspinal muscles in adolescent idiopathic scoliosis. Eur spine journal: official publication Eur Spine Soc Eur Spinal Deformity Soc Eur Sect Cerv Spine Res Soc.

[48] Wong C (2015). Mechanism of right thoracic adolescent idiopathic scoliosis at risk for progression; a unifying pathway of development by normal growth and imbalance. Scoliosis.

[49] Hamrick MW, McGee-Lawrence ME, Frechette DM (2016). Fatty Infiltration of Skeletal Muscle: Mechanisms and Comparisons with Bone Marrow Adiposity. Front Endocrinol.

[50] Kader DF, Wardlaw D, Smith FW (2000). Correlation between the MRI changes in the lumbar multifidus muscles and leg pain. Clin Radiol.

[51] Schleifer J, Fenzl G, Wolf A, Diehl K (1994). [Treatment of lumbar facet joint syndrome by CT-guided infiltration of the intervertebral joints]. Radiologe.

[52] Chen YY, Pao JL, Liaw CK, Hsu WL, Yang RS (2014). Image changes of paraspinal muscles and clinical correlations in patients with unilateral lumbar spinal stenosis. Eur spine journal: official publication Eur Spine Soc Eur Spinal Deformity Soc Eur Sect Cerv Spine Res Soc.

[53] Komolka K, Albrecht E, Wimmers K, Michal JJ, Maak S (2014). Molecular heterogeneities of adipose depots - potential effects on adipose-muscle cross-talk in humans, mice and farm animals. J genomics.

[54] Uezumi A, Fukada S, Yamamoto N, Takeda S, Tsuchida K (2010). Mesenchymal progenitors distinct from satellite cells contribute to ectopic fat cell formation in skeletal muscle. Nat Cell Biol.

[55] Joe AW, Yi L, Natarajan A, Le Grand F, So L, Wang J, Rudnicki MA, Rossi FM (2010). Muscle injury activates resident fibro/adipogenic progenitors that facilitate myogenesis. Nat Cell Biol.

